# A Comprehensive Assessment of Culturally and Linguistically Appropriate Emergency Preparedness and Crisis Response for Ohio’s Resettled Communities: A Mixed-Methods Assessment of Knowledge, Barriers, and Solutions

**DOI:** 10.3390/ijerph22101516

**Published:** 2025-10-02

**Authors:** Isha Karmacharya, Surendra Bir Adhikari, Elizabeth Poprocki, Mary Neely Young, Shuayb Jama, Denise Martin, Seleshi Ayalew Asfaw, Saruna Ghimire

**Affiliations:** 1 Department of Sociology and Gerontology and Scripps Gerontology Center, Miami University, Oxford, OH 45056, USA; karmaci@miamioh.edu; 2 Ohio Department of Mental Health & Addiction Services, Columbus, OH 43215, USA; surendra.adhikari@mha.ohio.gov (S.B.A.); elizabeth.poprocki@mha.ohio.gov (E.P.); 3 Department of Kinesiology, Nutrition, & Health, Miami University, Oxford, OH 45056, USA; youngm5@miamioh.edu; 4 Ethiopian Tewahedo Social Services, Columbus, OH 43213, USA; jet.jama@ethiotss.org (S.J.); denise.martin@ethiotss.org (D.M.); seleshi.asfaw@ethiotss.org (S.A.A.)

**Keywords:** refugee health, emergency preparedness, public health emergencies, youth engagement

## Abstract

This study evaluated emergency preparedness, covering knowledge, challenges, and culturally and linguistically appropriate resources among five resettled refugee communities in Central Ohio: Afghan, Bhutanese, Congolese, Ethiopian/Eritrean, and Somali. It also explored youth perceptions of community engagement and the effectiveness of current crisis support initiatives. A mixed-methods approach was used, consisting of a cross-sectional quantitative survey of 266 adults and a qualitative 60-min focus group discussion with 10 youth from the resettled communities. Quantitative data was analyzed with descriptive statistics and Chi-square tests assessing differences in preparedness across communities. Quantitative findings showed that the Bhutanese group reported the highest familiarity with preparedness concepts (33.3%), while Afghan and Congolese communities showed significant gaps. Of the 266 overall respondents, only 39.5% had an emergency plan, and 15.8% felt extremely confident in handling emergencies. Language barriers were major challenges, along with those related to race and ethnic identity, religious practices, and cultural norms. Effective platforms for engagement included community meetings, religious sites, and social media, with text messages and phone calls preferred for emergency communication. Youth discussion highlighted key public health concerns, particularly gun violence, substance abuse, and mental health crises, with stigma and limited resources further hindering preparedness efforts. Participants emphasized the need for culturally relevant interventions and stronger community engagement.

## 1. Introduction

An emergency is a sudden, unexpected situation that poses an immediate risk to life, property, or the environment, such as a medical crisis, and requires urgent action, but is usually manageable with local resources [1]. A disaster, on the other hand, is a large-scale catastrophic event that overwhelms local capacity and necessitates external assistance. While both involve critical threats, disasters are broader in scale and impact than emergencies [1]. Although disaster and emergency situations can affect anyone, a group that remains particularly vulnerable to their consequences is refugees. The global migration crisis has triggered unprecedented numbers of refugees and asylum seekers pursuing refuge across international borders, often escaping war, violence, and political persecution [2]. Since the enactment of the Refugee Act in 1980, the United States has granted refuge to over 3.8 million refugees and asylees [3]. As political tensions and persecution continue to marginalize groups, it is estimated that roughly 2.9 million refugees will need resettlement in 2025, an increase of half a million individuals compared to 2024 [4]. Ohio has been a leading state for refugee resettlement, ranking among the top five nationally between 2016 and 2018 [5]. From 2010 to 2021, over 25,000 refugees were officially resettled in Ohio [5], though actual numbers may be higher due to secondary inter-state migration and unofficial arrivals. In Central Ohio, the largest refugee groups originate from Somalia, Bhutan, the Democratic Republic of Congo, Iraq, Afghanistan, and Myanmar [6].

Although resettled communities in the United States are provided safety and sanctuary, they often face challenges accessing and navigating basic services due to language barriers, cultural differences, limited resources, and systemic inequities. These factors also impact their ability to prepare for and respond to public health emergencies [7]. Emergencies and disasters—whether natural (e.g., earthquakes, floods) or man-made (e.g., chemical spills, terrorism)—are high-impact events that disrupt daily life and require immediate, coordinated responses to reduce human, social, and economic harm [8]. Public health emergencies threaten population health, including disease outbreaks and environmental disasters [9]. Emergencies and disasters have disproportionately impacted racial and ethnic minorities, marginalized groups, immigrants, and refugees due to underlying social vulnerabilities, systemic inequities, and barriers to accessing resources and services [7,10]. Moreover, substance abuse and gun violence, recognized as a chronic, man-made disaster, mirror the social and psychological impacts of disasters in resettled communities already facing socioeconomic stress and systemic marginalization [11].

Research has shown that trauma exposure—especially through direct or witnessed violence—is a major risk factor for developing behavioral health disorders such as substance abuse, depression, anxiety, and post-traumatic stress disorder [12]. Emergencies often amplify these risks by intensifying pre-existing vulnerabilities and introducing new stressors. Refugees already coping with the complex challenges of displacement and resettlement face even greater mental health burdens when public health emergencies or other crises occur [13,14]. Moreover, access to mental health care is often restricted due to multiple barriers, including legal and language limitations, discrimination, and healthcare shortages [14]. Disaster studies have consistently shown that racial and ethnic minority communities in the United States are more vulnerable to both the immediate and long-term impacts of emergencies due to systemic inequalities. These impacts extend beyond mental health outcomes. Resettled refugees frequently experience immediate challenges during disasters such as job loss, housing instability, food insecurity, and limited access to healthcare, vaccination, and public services [10]. In the long term, these stressors can lead to chronic poverty, reduced educational attainment, persistent health disparities, and structural exclusion from emergency response systems, particularly for those with precarious legal status or language barriers [10].

The COVID-19 pandemic further accentuated health disparities, with marginalized populations experiencing higher rates of infection and mortality [15]. In its aftermath, resettled communities faced a myriad of overlapping challenges, including increased behavioral health issues, substance use, traumatic events, and domestic violence [16,17]. These pre-existing conditions and stressors may increase vulnerability during disasters and emergencies by exacerbating stress responses and limiting coping capacity. For example, the prevalence of depression among refugees is estimated to be twice as high as that of the general United States population, while post-traumatic stress disorder rates in some groups, such as Syrian, Iraqi, and Sudanese refugees, have reached as high as 40% [18]. Such elevated rates of mental health conditions may further worsen during emergencies, constraining help-seeking behaviors and disaster resilience, and thereby increasing vulnerabilities for these communities.

Given that refugee youth are vital resources within their communities, it may also be important to understand their perspectives and the essential roles they could play in developing culturally responsive and effective emergency preparedness strategies. Due to their relatively higher fluency in the host country’s language, digital literacy, and integration into educational and civic networks, youth often serve as cultural and linguistic brokers between their families and external systems. Research has demonstrated the instrumental effects of young people during crisis response, with them taking on roles like translating official disaster information, relaying critical safety messages, and supporting community recovery through their social networks [19]. The duality of refugee youth anchors them in both their cultural communities and the broader host society, uniquely equipping them to navigate and disseminate emergency information. Despite these capacities, youth are frequently overlooked in emergency planning [19].

Despite decades of research on public health emergencies, vulnerable populations, including racial and ethnic minorities, refugee populations, etc., continue to face systemic barriers to preparedness, response, and recovery. Nationally, the threat and hazard landscape is rapidly evolving. According to the Federal Emergency Management Agency’s (FEMA) 2023 National Preparedness Report, climate-related disasters are becoming more frequent, severe, and costly, with 60 major climate events causing over 1400 fatalities between 2020 and 2022 alone [20]. Historical events such as Hurricane Katrina revealed the devastating consequences of failing to account for cultural, linguistic, and socioeconomic diversity in emergency planning [21]. Poor and racially minoritized communities, especially those lacking transportation and clear communication, suffered disproportionately from the hurricane’s aftermath [21]. Furthermore, in many high-risk communities, critical response capabilities, such as sheltering, housing, and logistics, are significantly underfunded and underdeveloped [20].

Developing the capacity of resettled communities in emergency planning is vital for equitable crisis response, yet gaps remain in understanding their access to and use of services during emergencies and disasters. Key barriers include a lack of awareness and culturally or linguistically appropriate support [22]. To ensure equitable healthcare access and human service programs, targeted outreach strategies are essential [23]. Assessing resettled people’s knowledge and service utilization can help to identify gaps and unmet needs, fostering their involvement in preparedness and response efforts.

The primary aim of this study was to assess the prevalent structure of emergency preparedness within resettled refugee communities in Ohio, identify their knowledge and awareness, specific needs and challenges, and evaluate the availability and adequacy of culturally and linguistically appropriate emergency response information and support services. Additionally, this study pursued a qualitative approach to gauge the perceptions and experiences of youth regarding the extent of community engagement and participation in emergency preparedness activities as well as the effectiveness of current emergency preparedness initiatives.

## 2. Materials and Methods

### 2.1. Study Design

This study utilized a mixed-methods design, combining quantitative and qualitative approaches to gain a comprehensive understanding of public health crises, emergency preparedness experiences, and the challenges faced by resettled refugee communities in Ohio. It focused on five refugee groups from Afghanistan, Bhutan, Congo, Ethiopia/Eritrea, and Somalia. The quantitative component involved a cross-sectional survey of 266 adults, while the qualitative component included a focus group discussion exploring the perceptions and experiences of youths. This study employed a convergent parallel mixed-methods design, in which quantitative and qualitative data were collected simultaneously and then integrated during interpretation to provide a more comprehensive understanding of the findings.

### 2.2. Quantitative Survey

A cross-sectional survey was administered to resettled adults who met the following eligibility criteria: aged 18 years or older, residing in Ohio for at least the past six months, and having a refugee background from one of the five selected communities.

#### 2.2.1. Sample Size and Sampling

The sample size was calculated using a standard formula for estimating proportions: n = Z^2^ p (1 − p)/e^2^, where n represents the required sample size, Z is the Z-score corresponding to the desired confidence level (1.96 for a 95% confidence interval), p is the estimated proportion of the population with the characteristic of interest, and e is the margin of error. Based on the 2023 annual National Household Survey on Disaster Preparedness conducted by FEMA [20], which reported that 45% of United States adults were prepared for a disaster, we used p = 0.45 and 1 − p = 0.55 with a 6% margin of error, yielding a calculated sample size of 266 participants.

Multiple strategies were pursued to recruit potential participants for the self-administered survey. First, we collaborated with local community-based and faith-based organizations within the selected communities to distribute survey flyers. Second, Ethiopian Tewahedo Social Services (ETSS), a well-established non-profit known for serving underserved refugee and new immigrant communities in Ohio, leveraged its connections to facilitate direct outreach to local organizations and potential participants. Third, faith-based institutions were engaged to distribute survey flyers during religious and cultural events. Finally, ETSS and local community-based organizations printed paper copies of the survey and utilized community navigators to distribute them to community members who preferred a paper-based format.

#### 2.2.2. Data Collection 

Survey data were collected anonymously using both digital and paper formats. Participants could complete the survey via Qualtrics [24], an online survey platform, or through a paper-based version distributed by community leaders and ETSS’ Community Mental Health Navigators. For participants using the paper-based format who had limited literacy, community leaders assisted by reading the questions aloud and recording their responses. To transcend the cultural and linguistic barriers, the survey was available in eight languages: English, Amharic, Dari, Lingala, Nepali, Pashto, Somali, and Swahili. The survey tool was adapted from the FEMA National Household Survey on Disaster Preparedness [25].

#### 2.2.3. Study Measures and Variables

##### Socio-Demographics

The survey included select socio-demographic variables, such as community affiliation, age, gender, length of residence, educational attainment, English language proficiency, household composition, and disability status. Participants self-identified their community affiliation from a list including Afghan, Bhutanese, Congolese, Ethiopian/Eritrean, and Somali. Age was measured as the number of completed years and recoded into four groups: 18–25, 26–35, 36–45, and 45 years or older. Gender identity was assessed by asking participants to select from “male,” “female,” or “non-binary/third gender.” Length of residence in Ohio was measured using three categories: less than 1 year, 1–5 years, and more than 5 years. Educational attainment was categorized as no formal education, less than high school, high school graduate or equivalent, and college degree; the first two categories were combined due to small cell sizes. English language proficiency was assessed through three separate binary items indicating whether the participant could read, write, or speak in English. Household composition was measured as two continuous variables: the number of adults and the number of children under 18 residing in the household. Disability status was measured using two binary (yes/no) items: whether the participant had a disability and whether they were caring for someone with a disability.

##### Emergency Preparedness Knowledge and Emergency Experiences

Participants’ familiarity with emergency preparedness concepts was assessed using a single Likert-scale item with four response options: not familiar at all, slightly familiar, somewhat familiar, and very familiar. A follow-up question asked participants to indicate the specific types of emergency preparedness information they were familiar with, using a structured checklist that allowed multiple responses. Response options included: gathering or updating emergency supplies, insuring property or belongings, getting involved in community efforts, learning evacuation routes and plans, making an emergency plan, making the home safer, planning with neighbors for emergencies, and practicing emergency drills, among others (See Appendix A Table A1). The survey also provided an open-ended option for participants to specify any additional types of information not captured in the predefined list.

Disaster experience in the United States was measured with a binary (yes/no) item asking whether participants had personally experienced a disaster or emergency since resettling in the United States. To draw specific insights on these experiences, participants were asked to respond to what type of events (e.g., home fire, hurricane, flood, pandemic, extreme heat) they had encountered in their lifetime. Each event was treated as a separate binary variable. An open-ended “other” option was included to capture additional experiences.

##### Perceived Risk, Confidence, and Emergency Plans

Perception of disaster risk was measured by asking participants whether they believed a disaster was likely to occur in their area, using a three-point Likert scale with the response options: likely, unlikely, or don’t know. Confidence in disaster preparedness—specifically, how prepared they felt if a disaster were to occur—was assessed using a four-point scale: not confident at all, somewhat confident, moderately confident, and extremely confident.

Participants were also asked whether they had an emergency plan, with responses recorded as yes or no. Those who self-reported affirmatively were asked to specify the components of their plan using a structured checklist. Response options included actions such as gathering or updating emergency supplies, protecting important documents, and saving money for emergencies, among others (See Appendix A Table A1). An open-ended “other” option was provided to capture any additional planning activities beyond the predefined list.

##### Challenges in Accessing Emergency Preparedness Services

Challenges in accessing emergency assistance were assessed using a single item with three response options: yes, no, or never needed. Knowledge of where to seek emergency assistance when needed was measured using a binary (yes/no) question, indicating whether participants knew where to go for help during an emergency. Participants were also asked whether they had experienced language barriers when trying to access emergency-related services, with responses recorded as yes or no. Perceptions of the cultural appropriateness of available emergency preparedness materials were assessed using a three-point scale: very appropriate, somewhat appropriate, or not appropriate at all. To identify other service-related barriers, participants were presented with a checklist of potential challenges, including racial or ethnic discrimination and cultural or religious mismatches. Each of these items was measured as a separate binary (yes/no) variable. An open-ended “other” option allowed participants to report any other barriers not listed.

Participants were also asked whether they had participated in any formal emergency preparedness training within the past one to three years, with a binary (yes/no) response. Those who had not participated were asked to identify specific challenges that may have prevented their participation. Response options included lack of awareness of available training, training not offered in their language, scheduling conflicts, lack of culturally competent staff or interpreters, and incongruence with religious or cultural practices. An open-ended “other” option was provided for participants to specify any additional challenges not listed.

##### Community Engagement and Strategies for Community-Level Preparedness

A binary item asked whether the participant’s community had ever engaged in discussions about emergency preparedness, with response options of yes or no. To assess preferences for community engagement, participants were asked to select the most effective methods for discussing emergency preparedness. Multiple options were provided, including community meetings, gatherings at places of worship (e.g., churches or mosques), social media campaigns, and partnerships with community-based organizations. Participants’ preferences for communication tools to support emergency preparedness were also measured using a checklist that included emails, text messages or phone calls, flyers, storybooks, games, websites or apps, lesson plans, and activity books. Participants were allowed to select multiple preferred methods and tools.

In addition, participants were asked to identify strategies they believed would improve emergency preparedness efforts within their communities. These strategies were assessed using a multiple-choice format with binary (yes/no) responses for each item. Options included the creation of community-generated materials in local languages, multilingual preparedness training, and multilingual staff hired from local communities. An open-ended “other” option was provided in all multiple-choice questions to allow participants to specify additional methods or strategies not captured in the predefined list.

#### 2.2.4. Data Analyses 

Information from paper-based surveys was entered into Qualtrics to streamline the data analysis process. Next, these survey responses were exported from Qualtrics (Version 2020, Qualtrics International Inc., Provo, UT, USA) to SAS statistical software (Version 9.4, SAS Institute Inc., Cary, NC, USA) for analysis [26]. Descriptive statistics were used to summarize the data, with means, medians, and standard deviations calculated for continuous variables, and frequencies and percentages for categorical variables. To explore differences across the five communities, we conducted Chi-square tests, with statistical significance set at *p* < 0.05. Complete case analysis was used to handle missing data.

### 2.3. Qualitative Study

The qualitative component of the study used a descriptive approach to explore youth perceptions and experiences related to public health emergencies and crises, emergency preparedness, and the effectiveness of existing resources and initiatives. This methodology followed the Consolidated Criteria for Reporting Qualitative Research guidelines, a standard checklist designed to ensure the credibility and trustworthiness of qualitative research [27].

#### 2.3.1. Sampling Strategy and Participant Selection

Participants for the qualitative study were selected purposively from the ETSS Youth Advisory Panel, which included representatives from each of the five refugee communities. Eligibility criteria required participants to be between 18 and 25 years old, have a refugee background from one of the selected communities, and be proficient in English. From this panel, ten youths (two from each community) were selected to ensure diverse representation and solicit a broad range of perspectives.

#### 2.3.2. Data Collection 

Data were collected through focus group discussion (FGD) to encourage dialogue and capture collective insights [28]. The FGD lasted approximately 60 min and was conducted in English at ETSS. An experienced facilitator from the research team led the discussion, while a note-taker documented observational field notes. The session was audio-recorded and subsequently transcribed verbatim, and analyzed using thematic analysis [29].

#### 2.3.3. Study Tool

The FGD guide was developed through an extensive review of the literature and consultations with the community leaders from the five participating groups. The guide included open-ended questions and probing prompts to facilitate a more natural and inquiry-based conversation [30].

#### 2.3.4. Data Analyses

Dedoose Version 9.0.54 [31] was used to analyze the qualitative data, using thematic analysis, and following Braun and Clarke’s six-phase methodology [29]. The thematic analysis process involved identifying key ideas and concepts, generating initial codes, searching for themes, and reviewing and refining those themes. To ensure precision and clarity, an iterative process was followed to develop definitions for each code, category, and subcategory. Coding was performed independently by members of the research team, and discrepancies were resolved through consensus discussions to ensure consistency and reliability. Key themes and subthemes were derived inductively from the data and aligned with the study’s objectives. Field notes provided additional contextual insights, and participant quotes were used to highlight key themes and ideas.

## 3. Results

### 3.1. Quantitative Findings

#### 3.1.1. Socio-Demographic Characteristics of Survey Participants

The survey included 266 participants from five resettled communities, with relatively equal representation across groups, with a slightly higher representation of the Somali (22.2%) and Congolese (20.7%) communities. The majority (59%) of the participants were between the ages of 26 and 45. Women made up a higher proportion of the sample (54.5%) compared to men (43.6%) and non-binary/third gender groups (1.9%). More than half of the participants (56.2%) had lived in Ohio for over five years. In terms of education, 37.7% completed high school, while 32.5% held college degrees. Most participants reported a good level of English proficiency, with approximately 81% on average indicating they could read, write, or speak English, while 12.8% reported having no English proficiency. About one in ten participants reported having a disability (9.4%), while 15% lived with or cared for someone with a disability (Table 1).

#### 3.1.2. Emergency Preparedness Knowledge and Emergency Experiences

Participants’ knowledge about emergency preparedness varied across communities, with 22.6% of respondents being unfamiliar and 21.4% very familiar with key concepts (Table 2). The Bhutanese community exhibited the highest familiarity (33.3%), while Congolese respondents had the lowest (41.8%). On average, 20.7% reported experiencing emergencies in the United States. However, self-reported emergency experiences were varied across communities, with the Bhutanese group reporting the most experiences (52.9%), followed by Somali (30.5%). The common disasters and emergencies reported included pandemic, home fires, power outages, financial crises, severe storms, extreme heat, and floods (Appendix A Table A1).

#### 3.1.3. Perceived Disaster Risk, Preparedness Confidence, and Barriers to Emergency Assistance

Regarding perceived disaster risk, 31.6% believed a disaster was likely in their area (Table 2). Confidence in disaster preparedness was generally low, with only 15.8% of respondents feeling extremely confident. Somali respondents were the most confident (20.3%), while Afghan participants were the least (12.2%) (Table 2). Family emergency plans in general were limited across communities, with only 39.5% of respondents having one. Bhutanese participants were the most likely to have a plan (60.8%), while Congolese respondents were the least likely (27.3%). Although 40% of respondents never needed emergency assistance, 51.5% reported that they knew where to access help if needed, with Bhutanese respondents being the most confident (72.5%) and Ethiopian/Eritrean respondents the least (42.3%).

In terms of access to emergency assistance, language barrier (42.3%) was reported to be the most pronounced challenge, particularly for Bhutanese (52.9%) (Table 2). In response to another related question, respondents also reported other challenges, such as race, gender, religious practices, and cultural norms, with Congolese (28.6%) and Somali (21.4%) respondents reporting the highest race-related challenges (Appendix A Table A1).

#### 3.1.4. Emergency Preparedness Materials, Training, and Community Discussions

Perceptions around the cultural relevance of preparedness materials varied widely. While 30.3% found the materials “very appropriate,” 31.1% found them “not at all appropriate.” While the Bhutanese (43.1%) and Afghan (38.3%) respondents viewed the materials most favorably, Congolese (47.3%) and Somali (44.1%) respondents found them least culturally appropriate (Table 2). Participation in preparedness training was low across all groups, with only 17.8% attending training in the past 1–3 years (Appendix A Table A1). Somali (23.7%) and Ethiopian/Eritrean (21.2%) respondents reported slightly higher participation. The main barrier to accessing preparedness training was a lack of awareness about available training (54.1%), followed by a lack of time to attend training (18.8%). About 12% reported training not offered in their language, particularly for Afghan and Congolese respondents (32.3% each, Appendix A Table A1). Additionally, only 28.6% of respondents reported community discussions about emergency preparedness, with the Bhutanese group leading these discussions (39.2%) and Ethiopian/Eritrean respondents reporting the least engagement (17.3%, Table 2). Community meetings were reported as the most effective methods for engaging communities in emergency preparedness (68.8%), followed by gatherings at faith-based sites (58.3%).

### 3.2. Qualitative Findings from Focus Group Discussion with Youths

#### Participant Characteristics

The qualitative FGD included 10 young adults (mean age: 20.1 years) representing five selected communities. The group consisted of six male and four female participants who shared their perceptions, experiences, challenges, and recommendations related to emergency preparedness and crisis response in their communities. Their responses centered on four main themes: (1) concerns about public health emergencies and crises, (2) awareness of existing resources, (3) challenges in emergency preparedness, and (4) pragmatic feedback and recommendations.

**Theme** **1.** *Public Health Emergencies and Crisis Concerns.*

Participants reported experiencing various public health emergencies within their communities, including substance abuse, gun violence, suicide, health crises such as the COVID-19 pandemic, and social isolation. They also highlighted community-specific concerns: gun violence in the Somali community, suicide in the Bhutanese community, and social isolation among women in the Afghan community.

*I think drug abuse is probably one of the biggest emergencies that I’ve seen in my community, especially among kids around my age, high school kids, and even, like, middle school kids… I wanted to add that I think for the Bhutanese community, suicide is also another huge issue that we’ve seen recently.*

(Bhutanese youth)

*Maybe gun violence, I would say, for my community…I would say that’s been a pretty big issue.*

(Somali youth)

*A lot of women, they don’t know how to speak [English], how to go outside. They are still at home… Like they’re saying, “I don’t want to go outside. I’m scared of everything [indicating social isolation].”*

(Afghan youth)

**Theme** **2.** *Availability and Access to Existing Emergency Preparedness Resources.*

Participants identified web search engines like Google and social media as their primary resources during emergencies or when seeking emergency hotlines. One participant emphasized the accessibility of these tools, stating, “*It’s literally a tap away… the main source we have for assistance is our phone*” (Somali youth; all participants nodded in agreement). Social service organizations and religious institutions were also recognized as important points of contact for emergency preparedness and crisis response. These organizations offer various support services, including English classes, behavioral health-related conferences, and free clinics. One participant highlighted the central role of religious spaces during crises, explaining, “*If anything were to happen, like a big crisis—let’s say a tornado or a shooting—everyone would convene at the mosque to figure out the course of action*,” (Somali youth).

In addition to formal resources, participants emphasized the significance of cultural and community-driven support systems in managing emergencies. An Ethiopian youth described “*Mahbär*,” a traditional social network among Ethiopian adults that fosters mutual aid during times of crisis. This sense of community solidarity was viewed as a vital resource for providing practical and emotional support during emergencies.

“*I know the Ethiopian community, it’s not like the younger generation, but the adults, they have, it’s called Mahbär. It’s like a social, like a union kind of… when anything happens to one of the members in the society, they have a way to help.*”

(Ethiopian youth)

However, not all participants felt their communities were adequately equipped for emergency preparedness. Youth from the Somali and Congolese communities expressed concerns about a lack of organized resources, with one participant stating, “*the community did not have anything that would necessarily help for emergency preparedness.*” Highlighting a significant gap in existing resources that could leave certain communities unprepared and vulnerable, another participant noted that:

“*I never really heard anything necessarily from, like even the leaders of our community. I know we get together with certain things, but for [emergency] preparedness—nothing.*”

(Congolese youth)

Moreover, participants recognized that while resources may exist, accessing them remains a significant challenge due to limited outreach and communication. As one Somali youth explained, “*There are definitely resources out there somewhere, but the issue is that finding out about them is so difficult because there’s no marketing for them*.” This lack of visibility means that even well-established programs may go underutilized. Additionally, practical barriers such as transportation further limit access, particularly for older adults who may struggle to attend language classes and emergency-related workshops.

**Theme** **3.** *Barriers to Emergency Preparedness.*

Participants emphasized that many challenges faced by refugee communities reflect a “*universal experience*” (Somali youth) tied to the broader realities of immigration. Four key barriers hindering emergency preparedness were identified: (1) communication and technology barriers, (2) impact of external influences, (3) normalization of crises, and (4) stigma.

**Theme** **3.1.** *Communication and Technology Barriers.*

Language barriers emerged as a primary challenge across all communities, particularly in accessing and understanding critical health information during emergencies. A Somali youth described the confusion this creates: “*It caused widespread confusion due to those language barriers… When there was COVID, when they [healthcare professionals/social workers] were trying to explain things, they [community members] had a really difficult time grasping the information*.” Participants also noted that the inability to speak English puts their community members at a disadvantage when navigating public health crises and support systems. As a Congolese youth explained, “*The inability to speak English puts them at a disadvantage*.”

These challenges disproportionately affect older generations who face additional difficulties with technology. A Bhutanese youth reflected on this generational divide, stating, “*In the older generation, they have more problems with technical navigation than youngsters who grew up in America*.” Another Bhutanese participant elaborated on the risks older adults face during emergencies: “*The older generation probably can’t even call 911 properly because they don’t speak English… For us, we can search the internet and find information, but for the older generation, if they’re alone in an emergency and need to make a call themselves, they probably won’t know how to communicate or whom to go to*.” This sentiment was widely shared, as indicated by all participants nodding in agreement.

**Theme** **3.2.** *Impact of External Influences on Community Cohesion and Emergency Preparedness.*

Participants raised concerns about the growing influence of external sources, particularly social media, on their communities. They described how these influences are transforming their once-supportive, collective environments into more individualistic and fragmented spaces. This shift, they argued, has weakened the community’s ability to unite during emergencies, thereby hindering unified emergency preparedness.

A Somali youth reflected on these transformations, stating, “*Our community is very heavily influenced by outside sources quite frequently. We went from being…a community that always helps each other to grow and… persevere to becoming…a lot more selfish, turned towards gun violence and drug use.*” Another participant from the Ethiopian community echoed these concerns, emphasizing the role of social media in shaping attitudes and behaviors:

“*I feel like a lot of it has to do with social media. People see everything online and try to be like that, losing their true selves. I’ve seen friends change, becoming more violent, influenced by what they see, even though they weren’t like that when they were younger.*”

(Ethiopian youth)

Participants warned that this cultural shift away from collective responsibility could reduce the community’s ability to respond effectively to crises. However, they also recognized that external influences are not entirely negative. Some participants acknowledged that social media and other outside sources could promote positive outcomes, such as raising awareness about behavioral health or sharing emergency resources. This nuanced perspective reflects an understanding that external forces can both strengthen and weaken community resilience, depending on how they are engaged.

**Theme** **3.3.** *Normalization of Trauma and Crises.*

Participants highlighted how the normalization of public health crises—such as gun violence, substance abuse, and suicide—serves as a significant barrier to emergency preparedness. They explained that repeated exposure to traumatic events has desensitized many community members to view these crises as mere routine, diminishing the urgency to address the root causes and needed preventative efforts. This sense of normalization may hinder efforts to implement proactive emergency preparedness strategies, as crises are often treated as inevitable rather than preventable.

A Somali youth described this superficial approach to addressing crises, stating, “*They usually don’t really touch upon the root of the problem and kind of leave it at the surface*.” This pattern of overlooking the underlying causes of crises, participants argued, prevents communities from engaging in meaningful conversations about prevention and preparedness. The normalization of trauma was particularly evident in the context of gun violence. One Somali participant provided a powerful example:

“*Gun violence still happens to this day. I feel when someone passes away, the community kind of comes together, but we don’t talk about how we should stop this… We don’t talk about how we could move forward from this.*”

Participants also observed that many community members have become desensitized to trauma due to their backgrounds and lived experiences. A Bhutanese youth remarked:

*“People don’t question why it might have happened… And they’re so used to going through trauma because of the background they come from, they don’t even think of it as trauma.”*

**Theme** **3.4.** *Stigma Around Mental Health and Its Impact on Help-Seeking.*

Participants identified stigma—particularly surrounding mental health—as a significant barrier to seeking help and accessing available resources. This stigma is deeply ingrained in many refugee communities, leading individuals to suppress their struggles rather than seeking professional assistance. A Bhutanese youth described the isolating effects of this stigma: “*Mental health is not seen as a good thing. It’s really stigmatized. So, if you have some type of mental health problem, you’re literally just bottling it up until you explode one day*.”

This stigma was especially pronounced among older adults, who are often reluctant to seek mental health services due to fear of being judged or a lack of awareness about available resources. This reluctance can prevent individuals from receiving timely support, increasing their vulnerability during times of crisis.

**Theme** **4.** *Pragmatic Recommendations for Community-Centered Emergency Preparedness.*

Participants provided actionable suggestions to enhance emergency preparedness within their communities. Their recommendations fell into three key subthemes: (1) community engagement strategies, (2) improving communication and information dissemination, and (3) strengthening cultural and social support systems.

**Theme** **4.1.** *Community Engagement Strategies.*

Participants identified the critical role of community people, particularly community leaders and youths, as key contributors to community preparedness. They recommended that community leaders actively engage youths by assigning them specific roles in emergency preparedness initiatives. This could include mobilizing existing youth or student associations and collaborating with local social service organizations to build a more prepared and connected community. Participants proposed that regular community gatherings—such as cultural events, game nights, and youth-led workshops—would foster a sense of belonging while encouraging young people to take on leadership roles. They suggested that these activities could serve as entry points for discussing emergency preparedness in a more approachable and less formal manner.

“*They are more comfortable spending time together first; like for fun; and then going out together to volunteer or assist others in the community.*”

(Ethiopian youth)

Additionally, participants stressed that empowering youth to disseminate information and serve as cultural liaisons between their communities and external organizations could bridge generational and linguistic gaps in emergency planning.

**Theme** **4.2.** *Improving Communication and Information Dissemination.*

A major concern among participants was the limited awareness and accessibility of formal emergency resources, such as hotlines and crisis centers. They stressed that existing resources often fail to reach vulnerable populations due to language barriers, lack of visibility, and ineffective communication strategies. Participants recommended several practical solutions to improve information dissemination:

Translation Services: They called for providing multilingual emergency information, including translated versions of preparedness materials and access to interpreters for emergency assistance. One participant remarked, “*If 911 got some translators in a different language, that would help a lot*.”

(Congolese youth)

Community-Centered Messaging: Participants emphasized the importance of delivering information through trusted community figures and familiar platforms. One Bhutanese youth explained how community-specific communication can enhance trust and reach:

“*For my community, the person presenting the information in the video needs to be someone from within our community. It’s important that the video features someone familiar and trusted by the audience. Additionally, instead of solely relying on YouTube, this video should be shared across mandir [temple] pages, [local community organization] pages, and similar platforms, encouraging reposting. Especially on mandir pages, the older generation places a great deal of trust in spiritual leaders, so having their endorsement or involvement would make the message more impactful.*”

(Bhutanese youth)

Leveraging Social Media: Participants acknowledged the powerful role of social media in rapidly spreading critical information. One Somali youth reflected on how widespread media coverage during the COVID-19 pandemic increased their understanding:

“*I think the biggest resource was widespread outreach. Like obviously seeing all that chaos and all those news outlets going insane over this [COVID-19 pandemic crisis] and it being widely spread through TikTok, helped me better grasp the situation at large.*” (Somali youth)

Participants suggested that emergency preparedness messaging should be tailored for different platforms and audiences, targeting younger individuals through social media and older adults through religious institutions and in-person outreach.

**Theme** **4.3.** *Strengthening Cultural and Social Support Systems.*

Participants emphasized the vital role of cultural and faith-based support systems in their communities. They described how, during crises, many community members turn to religious institutions for guidance and collective action. One Somali youth highlighted the centrality of faith in crisis response: “The biggest types of interventions we usually have stemmed from religion.”

Faith-based centers, including mosques, temples, and churches, were seen not only as spiritual anchors but also as physical meeting points where the community could gather to organize and respond to emergencies. Participants recommended partnering with religious leaders to deliver emergency preparedness training and to facilitate ongoing dialogue about community safety. Additionally, participants recognized the value of external assistance and expressed openness to learning from outside organizations. An Afghan youth shared their enthusiasm for programs that offer practical skills and knowledge:

“*If organizations like yours or others offer similar programs for people like me, it can help us improve ourselves. I’ve learned a lot from them [other youths in FGD], and I think it’s a great idea.*”

## 4. Discussion

The study findings underscore significant disparities in emergency preparedness engagement among five resettled communities in Ohio. The familiarity with emergency concepts was very low, with considerable variation between communities. Most participants (60.5%) lacked family emergency plans, and language barriers were the predominant challenge (42.3%) in accessing emergency assistance. The focus group with youth identified community-specific concerns (substance abuse, gun violence, suicide), barriers to preparedness (language limitations, normalization of trauma, mental health stigma), and potential solutions focusing on youth engagement, multilingual resources, and leveraging faith-based networks. An important consideration in interpreting our findings is that the quantitative and qualitative components included different target populations. The survey focused on adults from resettled refugee communities, whereas the focus group engaged youth. While this distinction means that the study does not represent a single homogeneous sample, we intentionally used a convergent parallel mixed-methods design to capture complementary perspectives. Including both adult and youth voices allowed us to obtain a more comprehensive understanding of community preparedness needs and strengths.

### 4.1. Knowledge and Awareness of Emergency Preparedness and Disparities by Communities

The preparedness for disaster among these communities was low (15.8%), which was significantly lower than the general population (45%) [20]. Notably, the Bhutanese community exhibited the highest levels of engagement in preparedness discussions, familiarity with emergency preparedness concepts, and confidence in accessing emergency assistance. This community also reported the highest percentage of emergency and/or disaster experiences (52.9%), suggesting a potential link between awareness and direct exposure to emergencies and increased preparedness efforts [32]. The Afghan community reported having the least confidence in accessing emergency assistance. Concerns about immigration status, particularly for those with humanitarian parole or pending asylum cases, may discourage Afghans from engaging with official services [33].

A lack of awareness about formal emergency assistance, crisis hotlines, and preparedness programs was a major challenge among the resettled communities. Expanding translation services for emergency hotlines, providing technical training programs, and prioritizing accessible, linguistically diverse communication methods, particularly for older adults, could ensure that all community members can effectively navigate emergency situations [34].

### 4.2. Community Experiences, Needs, and Barriers for Emergency Preparedness

Resettled communities have commonly experienced emergencies such as the COVID-19 pandemic, home fires, power outages, financial crises, severe storms, extreme heat, and floods, which align with emergencies reported in Ohio [35]. Their awareness of certain disasters may also vary based on their country of origin. About 71% of participants reported no discussions on emergency preparedness in their community. Since social and community support is vital for resettled people to integrate into new societies [36], addressing this gap is crucial. Without strong networks and preparedness conversations, resettled individuals may struggle to access resources or understand local systems in emergencies [37]. It is important to create inclusive platforms and support networks to help them navigate both regular and emergency situations.

There were discrepancies between the quantitative and qualitative findings. While behavioral health emergencies emerged as a key concern in qualitative data, they did not figure in the quantitative results. This shows that qualitative research allowed participants an outlet to express nuanced, lived experiences that might not be fully captured through predefined survey questions [38]. FGD was specifically insightful because participants identified substance abuse, gun violence, suicide, and health crises such as social isolation as pressing concerns. This is reflective of the fact that, even more than emergency preparedness and crisis support issues, there are other germane and broader systemic challenges that impact both the physical and mental well-being of members of these communities. One possible explanation for the discrepancy between the quantitative and qualitative findings is that these behavioral health crises (e.g., gun violence, suicide) were not listed as specific survey options, although participants could mention them under the “other” category. Beyond this survey limitation, such physical and behavioral health issues may also stem from a combination of pre-migration trauma, resettlement stressors, and systemic barriers in their new environments. Resettled people may turn to substance abuse as a coping mechanism for unresolved trauma and the stress of adapting to a new culture [39]. Social isolation can stem from language barriers, cultural differences, and fractured social connections. These factors can worsen mental health issues, heightening the likelihood of substance abuse and increasing the risk of suicide [40]. Moreover, the recent statement by the United States Surgeon General, describing loneliness as an epidemic [41], emphasizes the seriousness of the problem and calls for immediate, coordinated efforts from both society and healthcare systems.

Significant barriers to emergency preparedness were reported, including communication and technology barriers, external influences, normalization of crises, and stigma. Study findings in both quantitative and qualitative studies indicated that community engagement in emergency preparedness discussions was low. While community-driven responses and social service organizations play a crucial role in crisis response, gaps in awareness, accessibility, and resource dissemination remain significant obstacles. Addressing these barriers requires a multifaceted approach. For example, the Los Angeles County Community Disaster Resilience initiative showcased the success of a community-driven approach in creating disaster preparedness strategies [42]. This effort brought together various stakeholders, including first responders, neighborhood watch groups, and faith-based organization representatives, to enhance resilience and emergency readiness [42]. Thus, emergency preparedness strategies can be strengthened through community engagement, improved communication, and culturally tailored interventions.

Language barriers emerged as a dominant challenge, particularly among older generations, who struggle with both English as well as digital literacy. The inability to effectively access, comprehend, and act on emergency-related information places them at higher risk during crises. These findings align with previous research emphasizing the importance of linguistically and culturally appropriate communication strategies for resettled communities [37]. They also reflect Uekusa’s concept of “disaster linguicism,” which highlights how linguistic minorities face disaster vulnerabilities due to language-based discrimination at both structural and interpersonal levels [43]. Uekusa’s work with immigrant and refugee communities in New Zealand and Japan shows how institutional practices privileging dominant languages restrict access to emergency information and services—paralleling our participants’ experiences with hotlines, preparedness materials, and crisis communication [43]. The persistence of English-only emergency protocols exemplifies structural discrimination and underscores the need for multilingual, culturally responsive strategies such as those recommended by our participants.

Participants highlighted concerns about the transformative effects of social media on their communities. While digital platforms serve as vital sources of emergency information, they also contribute to individualization and fragmentation, weakening traditional community-based support systems. This aligns with studies suggesting that increased digital engagement can alter social structures, shifting the reliance from collective resilience to more isolated, self-directed responses [44,45]. However, participants also acknowledged that social media can positively impact crisis awareness, particularly during global emergencies like the COVID-19 pandemic [46]. This dual perspective underscores the need to leverage social media as a tool for community-centered emergency preparedness while being mindful of its potential to disrupt traditional support networks.

The stigma surrounding mental health was a recurring emergency concern, particularly among older generations. These findings indicated that mental health issues are hidden emergencies that often go neglected. Many participants described how individuals within their communities suppress mental health struggles due to fear of judgment, which may contribute to delayed or entirely avoided help-seeking behaviors. This finding is consistent with studies on mental health stigma in immigrant and refugee populations, where cultural norms often frame mental health issues as personal weaknesses rather than medical conditions [47]. To address this, interventions must incorporate culturally sensitive education and trusted community leaders, such as religious figures, to normalize mental health discussions and encourage help-seeking behaviors.

### 4.3. Leveraging Youth and Community Engagement in Emergency Preparedness

This study highlights the crucial role of youth in leading community-based emergency preparedness efforts. Implementing structured youth leadership programs and strengthening partnerships with local organizations can further empower young individuals to serve as emergency preparedness ambassadors within their communities [37].

Another notable theme that emerged was the normalization of public health emergencies, where crises such as gun violence, suicide, and substance abuse are often accepted as inevitable rather than something preventable. Participants noted that societal discussions tend to focus on immediate causes of death rather than on how some of these could have been prevented or on examining underlying systemic factors, which may contribute to a lack of urgency in addressing preventable crises. Shifting the narrative toward preventative measures, policy advocacy, and community-driven solutions is essential to breaking this cycle [42,48].

Faith-based organizations and traditional community structures remain central pillars of crisis response in resettled communities. However, a notable gap in formal emergency preparedness programs within these community hubs suggests an opportunity for collaboration between emergency management agencies and local faith-based institutions. By integrating culturally relevant preparedness training into religious and community gatherings, organizations can bridge existing gaps and enhance trust in formal emergency response systems [42].

Overall, addressing both tangible emergency preparedness and underlying social and psychological stressors could enhance community resilience and crisis response. Future efforts should prioritize culturally responsive strategies that integrate mental health support, ensuring that preparedness initiatives are both comprehensive and inclusive.

### 4.4. Strengths and Limitations of the Study

This study employed a mixed-methods approach, integrating quantitative surveys and qualitative focus groups to provide both breadth and depth in understanding emergency preparedness among refugee communities. The inclusion of five distinct refugee groups allowed for comparative analysis, highlighting community-specific needs and differences. The youth focus group offered valuable insights into intergenerational differences in emergency preparedness knowledge and experiences, while collaboration with local community organizations ensured cultural relevance in study methods and tools, facilitating participant recruitment and trust-building. To enhance accessibility and linguistic inclusivity, surveys were translated into eight languages and administered in both digital and paper formats, reducing barriers to participation. While translation helps to reduce linguistic barriers, we acknowledge that it alone does not fully address cultural differences between the communities. Although focus groups were conducted in English, language exclusion was minimal, as youth (18–25 years) were largely proficient due to school exposure.

Despite its strengths, the study has some limitations. The reliance on self-reported data may introduce recall bias; however, this is likely minimal as participants were asked about their current knowledge, experiences, and the effectiveness of existing resources. There is also the potential for response bias, particularly the possibility that participants may have overreported socially desirable behaviors such as having an emergency supply kit. However, because this was an anonymous online survey completed independently, the likelihood of such bias may have been reduced, although it cannot be entirely ruled out. Additionally, the study provides a cross-sectional snapshot of emergency preparedness at a single point in time, limiting the ability to assess changes over time or draw causal inferences. Future research could benefit from a longitudinal design to track evolving preparedness levels. Design, implementation, and evaluations of culturally tailored emergency preparedness interventions, especially those involving youth and religious institutions, would provide valuable evidence for scaling effective approaches.

## 5. Conclusions

This study examined emergency preparedness in five resettled communities in Ohio, revealing gaps in preparedness, service availability, accessibility, and community engagement, with significant variations between the communities. The study found that while communities exhibited a certain level of awareness of emergency preparedness, actual preparedness actions, confidence, and participation were low. Barriers such as language, the cultural relevance of preparedness materials, and unfamiliarity with available resources and hotlines must be addressed through specific educational programs to ensure equitable access to emergency assistance. Moreover, broader social and cultural concerns, including concerns about public health crises such as substance abuse, gun violence, and suicide, as well as deeply ingrained mental health stigma and trauma normalization, necessitate community-specific, tailored interventions. The study also highlights the crucial role of youth as bridges within their communities and the influence of faith-based institutions as decision-making and response hubs, suggesting that these community assets should be leveraged in future preparedness initiatives.

Enhancing emergency preparedness among refugee communities requires a culturally responsive, community-driven approach. Based on our findings, we propose several key strategies to improve outreach, engagement, and effectiveness of preparedness efforts. First, emergency preparedness materials and training must be developed in multiple languages with culturally appropriate content to address the language barriers identified by respondents. Multilingual training programs should be implemented through trusted community platforms, such as faith-based and cultural organizations, to ensure accessibility and relevance. Given that many respondents identified places of worship as effective engagement hubs, religious leaders and institutions—identified as effective platforms for health promotion [49]—can be leveraged for preparedness initiatives, helping to build trust and encourage participation. Second, a community-based approach that actively involves local leaders and youth can strengthen local capacity, collective responsibility, and resilience. Youth, in particular, should be engaged as key stakeholders, leveraging their technological skills and cultural fluency to bridge generational divides. Finally, integrating behavioral health support into preparedness efforts is also essential, addressing the stigma surrounding suicide, substance use, and social isolation within these communities. Emergency plans should include strategies for managing behavioral health issues and promoting emotional resilience. Trauma-informed, culturally sensitive emergency response strategies can improve trust and participation. Further, emergency communications must be culturally authentic, especially when addressing sensitive topics like suicide. Utilizing trusted community figures and familiar platforms will maximize outreach and effectiveness. Strengthening partnerships between emergency management agencies and local organizations will be critical to ensuring sustainable, inclusive preparedness strategies.

## Figures and Tables

**Table 1:** Socio-Demographic Characteristics of Study Participants (n = 266).

Characteristics	n (%)
**Community Representation**	
Afghan	49 (18.4)
Bhutanese	51 (19.2)
Congolese	55 (20.7)
Ethiopian and/or Eritrean	52 (19.6)
Somali	59 (22.2)
**Age Group**	
18–25	63 (23.7)
26–35	76 (28.6)
36–45	80 (30.1)
45 and over	47 (17.7)
**Gender**	
Male	116 (43.6)
Female	145 (54.5)
Non-binary/Third gender	5 (1.9)
**Length of Residence in Ohio**	
Less than 1 year	52 (19.6)
1–5 years	64 (24.2)
More than 5 years	149 (56.2)
**Educational Attainment**	
No formal education/Less than high school	79 (29.8)
High school graduate or equivalent	100 (37.7)
College degree	86 (32.5)
**^1^** **English Proficiency**	
Able to read in English	218 (82.0)
Able to write in English	206 (77.4)
Able to speak English	220 (82.7)
No English proficiency	34 (12.8)
**Number of Adults in Household** (Mean ± SD)	2.94 ± 1.73
**Number of Children Under 18 in Household** (Mean ± SD)	1.81 ± 1.64
**Disability Status**	
Yes	25 (9.4)
No	241 (90.6)
**Caring for Someone with a Disability**	
Yes	40 (15.0)
No	226 (85.0)

^1^ Indicates responses from multiple-choice questions; hence, the percentages do not add up to 100%, and the frequency exceeds the total sample size.

**Table 2:** Emergency Preparedness (EP) Knowledge and Experience, and Barriers to Services by Community.

Characteristics	Overall	Afghan	Bhutanese	Congolese	Ethiopian and/or Eritrean	Somali	^1^ *p*-Value
	n (%)	n (%)	n (%)	n (%)	n (%)	n (%)	
**Emergency Preparedness Awareness**							**<0.001**
Not familiar at all	60 (22.6)	13 (26.5)	5 (9.8)	23 (41.8)	3 (5.8)	16 (27.1)	
Slightly familiar	79 (29.7)	19 (38.8)	19 (37.3)	13 (23.6)	14 (26.9)	14 (23.7)	
Somewhat familiar	70 (26.3)	15 (30.6)	10 (19.6)	11 (20.0)	19 (36.5)	15 (25.4)	
Very familiar	57 (21.4)	2 (4.1)	17 (33.3)	8 (14.5)	16 (30.8)	14 (23.7)	
**Disaster Experience in the US**							**<0.001**
Experienced	55 (20.7)	2 (4.1)	27 (52.9)	7 (12.7)	1 (1.9)	18 (30.5)	
Not experienced	211 (79.3)	47 (95.9)	24 (47.1)	48 (87.3)	51 (98.1)	41 (69.5)	
**Disaster Risk Perception**							**0.002**
Likely	84 (31.6)	17 (34.7)	20 (39.2)	11 (20.0)	15 (28.8)	21 (35.6)	
Unlikely	61 (22.9)	2 (4.1)	12 (23.5)	13 (23.6)	13 (25.0)	21 (35.6)	
I don’t know	121 (45.5)	30 (61.2)	19 (37.3)	31 (56.4)	24 (46.2)	17 (28.8)	
**Confidence in Disaster Preparation**							**0.036**
Extremely confident	42 (15.8)	6 (12.2)	7 (13.7)	9 (16.7)	8 (15.4)	12 (20.3)	
Moderately confident	56 (21.1)	7 (14.3)	19 (37.3)	10 (18.5)	11 (21.2)	9 (15.3)	
Somewhat confident	77 (29.1)	14 (28.6)	10 (19.6)	13 (24.1)	23 (44.2)	17 (28.8)	
Not confident at all	90 (34.0)	22 (44.9)	15 (29.4)	22 (40.7)	10 (19.2)	21 (35.6)	
**Emergency Plan**							**0.008**
Has a plan	105 (39.5)	19 (38.8)	31 (60.8)	15 (27.3)	18 (34.6)	22 (37.3)	
No plan	161 (60.5)	30 (61.2)	20 (39.2)	40 (72.7)	34 (65.4)	37 (62.7)	
**Challenges Accessing Emergency Assistance**							**<0.001**
Yes	29 (10.9)	6 (12.2)	14 (27.5)	5 (9.3)	2 (3.8)	2 (3.4)	
No	129 (48.7)	34 (69.4)	18 (35.3)	26 (48.1)	17 (32.7)	34 (57.6)	
Never Needed	107 (40.4)	9 (18.4)	19 (37.3)	23 (42.6)	33 (63.5)	23 (39.0)	
**Knowledge of Emergency Assistance**							**0.016**
Knows where to get help	137 (51.5)	24 (49.0)	37 (72.5)	28 (50.9)	22 (42.3)	26 (44.1)	
Does not know	129 (48.5)	25 (51.0)	14 (27.5)	27 (49.1)	30 (57.7)	33 (55.9)	
**Language Barriers in Service Access**							0.216
Yes	112 (42.3)	20 (41.7)	27 (52.9)	20 (36.4)	17 (32.7)	28 (47.5)	
No	153 (57.7)	28 (58.3)	24 (47.1)	35 (63.6)	35 (67.3)	31 (52.5)	
**Cultural Appropriateness of Emergency Preparedness Materials**							**0.003**
Very appropriate	80 (30.3)	18 (38.3)	22 (43.1)	12 (21.8)	15 (28.8)	13 (22.0)	
Somewhat appropriate	102 (38.6)	20 (42.6)	19 (37.3)	17 (30.9)	26 (50.0)	20 (33.9)	
Not appropriate at all	82 (31.1)	9 (19.1)	10 (19.6)	26 (47.3)	11 (21.2)	26 (44.1)	
**Community Discussions on EP**							0.144
Yes	76 (28.6)	12 (24.5)	20 (39.2)	16 (29.1)	9 (17.3)	19 (32.2)	
No	190 (71.4)	37 (75.5)	31 (60.8)	39 (70.9)	43 (82.7)	40 (67.8)	
**^2^ Most Effective Methods for Engaging Communities in EP**							
Community meetings	183 (68.8)	40 (81.6)	41 (80.4)	34 (61.8)	37 (71.2)	31 (52.5)	
Gatherings at places of worship (e.g., churches, mosques)	155 (58.3)	25 (51.0)	27 (52.9)	22 (40.0)	41 (78.8)	40 (67.8)	
Social media campaigns	125 (47.0)	15 (30.6)	35 (68.6)	22 (40.0)	28 (53.8)	25 (42.4)	
Partnerships with community organizations	109 (41.0)	15 (30.6)	26 (51.0)	21 (38.2)	29 (55.8)	18 (30.5)	

^1^*p*-values from a Chi-square test comparing differences in emergency preparedness across various communities. ^2^ Indicates responses from multiple-choice questions, so the percentages do not add up to 100%, and the frequency exceeds the total sample size; row percentages are presented for each community based on the number of responses to the item.

## Data Availability

The datasets generated and/or analyzed during this study are not publicly available due to privacy and confidentiality concerns. They contain sensitive information regarding individuals’ demographics, experiences, and physical and behavioral health, which must be protected to safeguard participant privacy and dignity. However, de-identified datasets can be made available upon reasonable request to the corresponding author, contingent on obtaining the necessary ethical approval.

## Abbreviations

The following abbreviations are used in this manuscript:

ETSS	Ethiopian Tewahedo Social Services
FEMA	Federal Emergency Management Agency
FGD	Focus Group Discussion

## Appendix A

**Table A1:** Emergency Preparedness (EP) Practices, Perceptions, and Challenges Across Diverse Refugee Communities.

Characteristic	Overall	Afghan	Bhutanese	Congolese	Ethiopian and/or Eritrean	Somali
	n (%)	n (%)	n (%)	n (%)	n (%)	n (%)
**Type of Information**						
Gather or update emergency supplies	124 (46.6)	18 (36.7)	24 (47.1)	24 (43.6)	29 (55.8)	29 (49.2)
Insure property/belongings	80 (30.1)	16 (32.7)	15 (29.4)	12 (21.8)	24 (46.2)	13 (22.0)
Get involved in community EP	54 (20.3)	3 (6.1)	17 (33.3)	9 (16.4)	15 (28.8)	10 (16.9)
Learn evacuation routes and plans	78 (29.3)	17 (34.7)	15 (29.4)	9 (16.4)	21 (40.4)	16 (27.1)
Make an emergency plan	101 (38.0)	7 (14.3)	21 (41.2)	16 (29.1)	30 (57.7)	27 (45.8)
Make your home safer	136 (51.1)	19 (38.8)	30 (58.8)	27 (49.1)	36 (69.2)	24 (40.7)
Plan with neighbors for emergencies	48 (18.0)	4 (8.2)	16 (31.4)	4 (7.3)	12 (23.1)	12 (20.3)
Practice emergency drills	63 (23.7)	6 (12.2)	10 (19.6)	8 (14.5)	23 (44.2)	16 (27.1)
Safeguard important documents	75 (28.2)	18 (36.7)	12 (23.5)	10 (18.2)	21 (40.4)	14 (23.7)
Save money for emergencies	97 (36.5)	9 (18.4)	14 (27.5)	21 (38.2)	30 (57.7)	23 (39.0)
Sign up for alerts and warnings	67 (25.2)	5 (10.2)	22 (43.1)	6 (10.9)	23 (44.2)	11 (18.6)
Test family EP communication plan	41 (15.4)	2 (4.1)	13 (25.5)	5 (9.1)	12 (23.1)	9 (15.3)
**Experienced Disasters or Emergencies**						
Active shooter situation	1 (0.4)	0 (0.0)	0 (0.0)	1 (100.0)	0 (0.0)	0 (0.0)
Home fire	16 (6.0)	0 (0.0)	5 (31.3)	1 (6.3)	1 (6.3)	9 (56.3)
Hurricane	5 (1.9)	1 (20.0)	3 (60.0)	1 (20.0)	0 (0.0)	0 (0.0)
Landslide	1 (0.4)	0 (0.0)	0 (0.0)	1 (100.0)	0 (0.0)	0 (0.0)
Pandemic	25 (9.4)	0 (0.0)	18 (72.0)	2 (8.0)	0 (0.0)	5 (20.0)
Power outage	9 (3.4)	1 (11.1)	4 (44.4)	1 (11.1)	0 (0.0)	3 (33.3)
Thunderstorm	4 (1.5)	1 (25.0)	0 (0.0)	0 (0.0)	0 (0.0)	3 (75.0)
Tornado	6 (2.3)	0 (0.0)	4 (66.7)	1 (16.7)	0 (0.0)	1 (16.7)
Tsunami	1 (0.4)	0 (0.0)	0 (0.0)	0 (0.0)	0 (0.0)	1 (100.0)
Typhoon	1 (0.4)	0 (0.0)	1 (100.0)	0 (0.0)	0 (0.0)	0 (0.0)
Utility interruption	4 (1.5)	0 (0.0)	0 (0.0)	1 (25.0)	0 (0.0)	3 (75.0)
Cyberattack	2 (0.8)	0 (0.0)	1 (50.0)	1 (50.0)	0 (0.0)	0 (0.0)
Drought	1 (0.4)	0 (0.0)	1 (100.0)	0 (0.0)	0 (0.0)	0 (0.0)
Earthquake	2 (0.8)	1 (50.0)	1 (50.0)	0 (0.0)	0 (0.0)	0 (0.0)
Extreme heat	7 (2.6)	1 (14.3)	2 (28.6)	1 (14.3)	0 (0.0)	3 (42.9)
Financial emergency	6 (2.3)	0 (0.0)	1 (16.7)	2 (33.3)	0 (0.0)	3 (50.0)
Flood	4 (1.5)	0 (0.0)	3 (75.0)	1 (25.0)	0 (0.0)	0 (0.0)
**Description of Emergency Plan**						
Gathered or updated supplies for emergencies	47 (17.7)	10 (21.3)	12 (25.5)	5 (10.6)	8 (17.0)	12 (25.5)
Protected important documents	48 (18.0)	5 (10.4)	15 (31.3)	7 (14.6)	11 (22.9)	10 (20.8)
Saved money for emergencies	38 (14.3)	1 (2.6)	13 (34.2)	8 (21.1)	6 (15.8)	10 (26.3)
Signed up for alerts and warnings	39 (14.7)	0 (0.0)	16 (41.0)	5 (12.8)	7 (17.9)	11 (28.2)
Tested my family communication plan	23 (8.6)	0 (0.0)	7 (30.4)	4 (17.4)	5 (21.7)	7 (30.4)
Insured my property	44 (16.5)	1 (2.3)	14 (31.8)	4 (9.1)	15 (34.1)	10 (22.7)
Planned for financial emergencies	45 (16.9)	2 (4.4)	13 (28.9)	7 (15.6)	12 (26.7)	11 (24.4)
Got involved in my community	28 (10.5)	0 (0.0)	13 (46.4)	2 (7.1)	6 (21.4)	7 (25.0)
Learned my evacuation routes	40 (15.0)	7 (17.5)	7 (17.5)	5 (12.5)	11 (27.5)	10 (25.0)
Made an emergency plan	30 (11.3)	2 (6.7)	5 (16.7)	7 (23.3)	8 (26.7)	8 (26.7)
Made my home safer	45 (16.9)	6 (13.3)	12 (26.7)	8 (17.8)	12 (26.7)	7 (15.6)
Planned with my neighbors	21 (7.9)	2 (9.5)	8 (38.1)	0 (0.0)	4 (19.0)	7 (33.3)
Practiced emergency drills	25 (9.4)	1 (4.0)	8 (32.0)	1 (4.0)	7 (28.0)	8 (32.0)
**^1^** **Challenges in Accessing EP Services**						
Race or ethnicity	28 (10.5)	4 (14.3)	5 (17.9)	8 (28.6)	5 (17.9)	6 (21.4)
Religious practices	26 (9.8)	3 (11.5)	8 (30.8)	6 (23.1)	2 (7.7)	7 (26.9)
Cultural traditions/norms	41 (15.4)	8 (19.5)	9 (22.0)	7 (17.1)	5 (12.2)	12 (29.3)
No difficulties	191 (71.8)	37 (19.4)	37 (19.4)	37 (19.4)	42 (22.0)	38 (19.9)
**Participated in Preparedness Training (Past 1–3 Years)**						
Yes	47 (17.8)	5 (10.2)	8 (15.7)	9 (17.0)	11 (21.2)	14 (23.7)
No	217 (82.2)	44 (89.8)	43 (84.3)	44 (83.0)	41 (78.8)	45 (76.3)
**^1^** **Challenges Accessing Preparedness Training**						
Unaware of available training	144 (54.1)	32 (22.2)	27 (18.8)	20 (13.9)	35 (24.3)	30 (20.8)
Training not offered in my language	31 (11.7)	10 (32.3)	2 (6.5)	10 (32.3)	3 (9.7)	6 (19.4)
Trainings didn’t fit cultural/religious practices	12 (4.5)	1 (8.3)	1 (8.3)	6 (50.0)	1 (8.3)	3 (25.0)
Lack of culturally competent staff/interpreters	13 (4.9)	1 (7.7)	3 (23.1)	4 (30.8)	-	5 (38.5)
No time to attend training	50 (18.8)	5 (10.0)	15 (30.0)	11 (22.0)	8 (16.0)	11 (22.0)
**^1^** **Helpful Tools for Discussing EP in The Community**						
Emails	144 (54.1)	28 (19.4)	31 (21.5)	36 (25.0)	33 (22.9)	16 (11.1)
Text messages or phone calls	186 (69.9)	32 (17.2)	41 (22.0)	31 (16.7)	44 (23.7)	38 (20.4)
Activity books	53 (19.9)	10 (18.9)	10 (18.9)	15 (28.3)	10 (18.9)	8 (15.1)
Digital resources (websites or apps)	85 (32.0)	16 (18.8)	15 (17.6)	12 (14.1)	23 (27.1)	19 (22.4)
Handouts or flyers	90 (33.8)	8 (8.9)	18 (20.0)	19 (21.1)	25 (27.8)	20 (22.2)
Lesson plans (curriculum)	72 (27.1)	20 (27.8)	12 (16.7)	13 (18.1)	15 (20.8)	12 (16.7)
Games	31 (11.7)	11 (35.5)	3 (9.7)	10 (32.3)	3 (9.7)	4 (12.9)
Storybooks	20 (7.5)	6 (30.0)	4 (20.0)	1 (5.0)	5 (25.0)	4 (20.0)
**^1^** **Strategies for Improving EP in Communities**						
Community-created materials in local languages	182 (68.4)	33 (18.1)	32 (17.6)	32 (17.6)	44 (24.2)	41 (22.5)
Multilingual preparedness training	159 (59.8)	24 (15.1)	36 (22.6)	26 (16.4)	39 (24.5)	34 (21.4)
Multilingual staff from local communities	145 (54.5)	23 (15.9)	36 (24.8)	22 (15.2)	32 (22.1)	32 (22.1)

^1^ Multiple choice responses.
